# Supplementary material for: A Metabolic Enhancer Protects against Diet-Induced Obesity and Liver Steatosis and Corrects a Pro-Atherogenic Serum Profile in Mice
Source: Nutrients. 2023 May 22;15(10):2410. doi: 10.3390/nu15102410 (PMC10221984; doi:10.3390/nu15102410)
Supplement: Supplementary file 1 [file nutrients-15-02410-s001.zip › nutrients-2364714-supplementary.pdf]

A metabolic enhancer protects against diet-induced obesity, liver steatosis and corrects a pro-atherogenic serum profile in mice

Khrystyna Platk[1,†], Paul F. Lebeau[1,†], Joshua P. Nederveen[2,†], Jae Hyun Byun[1], Melissa E. MacDonald[1], Jacqueline M. Bourgeois[3], Mark A. Tarnopolsky[2,4,] and Richard C. Austin[1,]**

1 Department of Medicine, Division of Nephrology, McMaster University, and The Research Institute of St. Joe's Hamilton, Hamilton, Ontario, Canada.
2 Department of Pediatrics, Faculty of Health Sciences, McMaster University Medical Centre (MUMC), Hamilton, Ontario, Canada
3 Department of Pathology and Molecular Medicine, Faculty of Health Sciences, McMaster University Medical Centre (MUMC), Hamilton, Ontario, Canada
4 Exerkine Corporation, MUMC, Hamilton, Ontario, Canada
* Correspondence: tarnopol@mcmaster.ca (M.A.T.); austinr@mcmaster.ca (R.C.A.); Tel.: +905-522-1155 x35175 (R.C.A.); Fax: 905-540-6589 (R.C.A.).
† These authors contributed equally to this work.

Supporting Table S1: List of Antibodies

Antibody	Manufacturer	Product Number	Antibody Concentration	Application	Antibody Dilution
β-actin	Sigma-Aldrich	A2228	2mg/mL	Immunoblot	1:5000
cCasp-1	Abcam	AB138483	1mg/mL	Immunoblot	1:500
CD36	Novus-Biologicals	NB400-144	1mg/mL	IHC	1:100, no retrieval
CHOP	Santa Cruz Biotechnology	SC-793	200ug/mL	Immunoblot	1:500
GRP78	BD Biosciences	610979	250ug/mL	Immunoblot	1:1000
				IF	1:1000
GRP94	Enzo Life Sciences	ADI-SPA-850	200ug/mL	Immunoblot	1:1000
				IF	1:100
Fibronectin	Abcam	Ab2413	200ug/mL	IF	1:100
LDLR	R and D Systems	AF2255	1mg/mL	IF	1:100
				IHC	1:100, HIER
sXBP1	Santa Cruz Biotechnology	SC-8015	200ug/mL	Immunoblot	1:1000
XBP1	Santa Cruz Biotechnology	SC-8015	200ug/mL	Immunoblot	1:1000

Supporting Table S2: List of Primers Used for qRT-PCR

Gene	Species	Forward	Reverse
ADRP	Mouse	TCTCAGGGGTGGTGGATAA	TCTACCAGCAGCTCCGACTT
ATF4	Mouse	ATGGCCGGCTATGGATGAT	CGAAGTCAAACTCTTTCAGATCCATT
Casp1	Mouse	TCCGCGGTTGAATCCTTTTCAGA	ACCACAATTGCTGTGTGTGCGCA
Casp3	Mouse	CCTCAGAGAGACATTCATGG	GCAGTAGTCGCCTCTGAAGA
Casp7	Mouse	GGACCGAGTGCCCACTTATC	TCGCTTTGTCGAAGTTCTTGTT
Casp9	Mouse	AGGAGGGACGAACACGTCT	CAAAGAAGGTTGCCCCAATCT
CHOP	Mouse	CTGCCTTTCACCTTGGAGAC	CGTTTCCTGGGGATGAGATA
FN1	Mouse	CGAGGTGACAGAGACCACAA	CTGGAGTCAAGCCAGACACA
GRP78	Mouse	GTCCTGCATCATCAGCGAAAG	GGTAGCCACATACTGAACACCA
HNF1α	Mouse	CTTCCTTCTTCATGCCAG	ACACGTCCCCATCTGAAG
IL1β	Mouse	GCACTACAGGCTCCGAGATGAAC	TTGTCGTTGCTTGGTTCTCCTTGT
IRE1α	Mouse	TGAAACACCCCTTCTTCTGG	CCTCCTTTTCTATTCGGTCACTT
PARP1	Mouse	GGAAAGGGATCTACTTTGCCG	TCGGGTCTCCCTGAGATGTG
PERK	Mouse	CCTTGGTTTCATCTAGCCTCA	ATCCAGGGAGGGGATGAT
PCSK9	Mouse	TGCAAAATCAAGGAGCATGGG	CAGGGAGCACATTGCATCC
SREBP1	Mouse	ACCCTGGTGAGTGGAGGGACCATCT TGG	CTTTGCTTCAGTGCCCACCACCAGGT CTTT
SREBP2	Mouse	GTGGAGCAGTCTCAACGTCA	TGGTAGGTCTCACCCAGGAG
sXBP1	Mouse	GAGTCCGCAGCAGGTG	GTGTCAGAGTCCATGGGA
TNFα	Mouse	CATGAGCACAGAAAGCATGATCCG	AAGCAGGAATGAGAAGAGGCTGAG
TGFβ	Mouse	CAACAATTCCTGGCGTTACCTTGG	GAAAGCCCTGTATTCCGTCTCCTT
18S	Mouse	GGACCAGAGCGAAAGCATTTGCC	TCAATCTCGGGTGGCTGAACGC

Supporting Table S3: Criteria Used to Determine Steatosis Grade

Steatosis Grade		
0	none	No affected cells
1	mild	Small clusters
2	moderate	Rare macroglubule
3	extensive	Numerous macroglubule

Supplemental Figures and Figure Legends

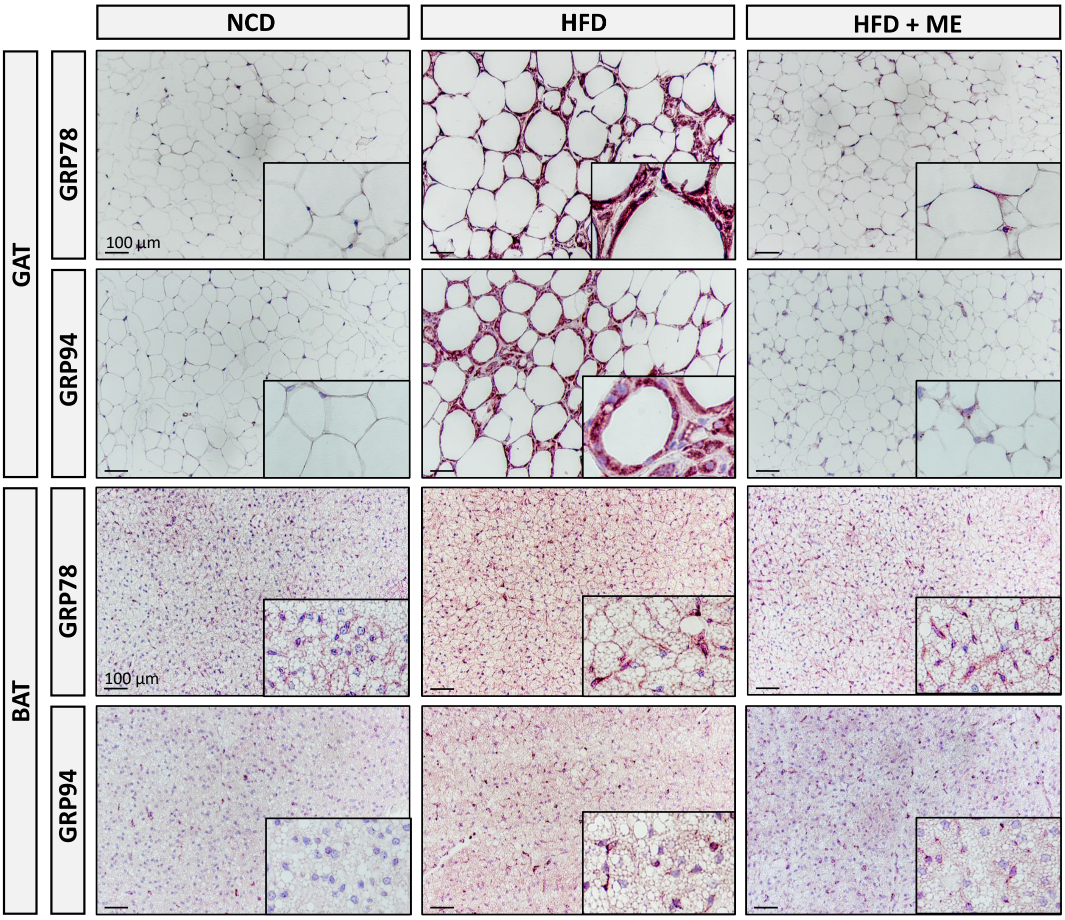

Supplemental Figure S1: Metabolic enhancer attenuates UPR activation in adipose tissue. Immunohistochemical staining of GRP78 and GRP94 in GAT and BAT. Scale bars, 100 µm.

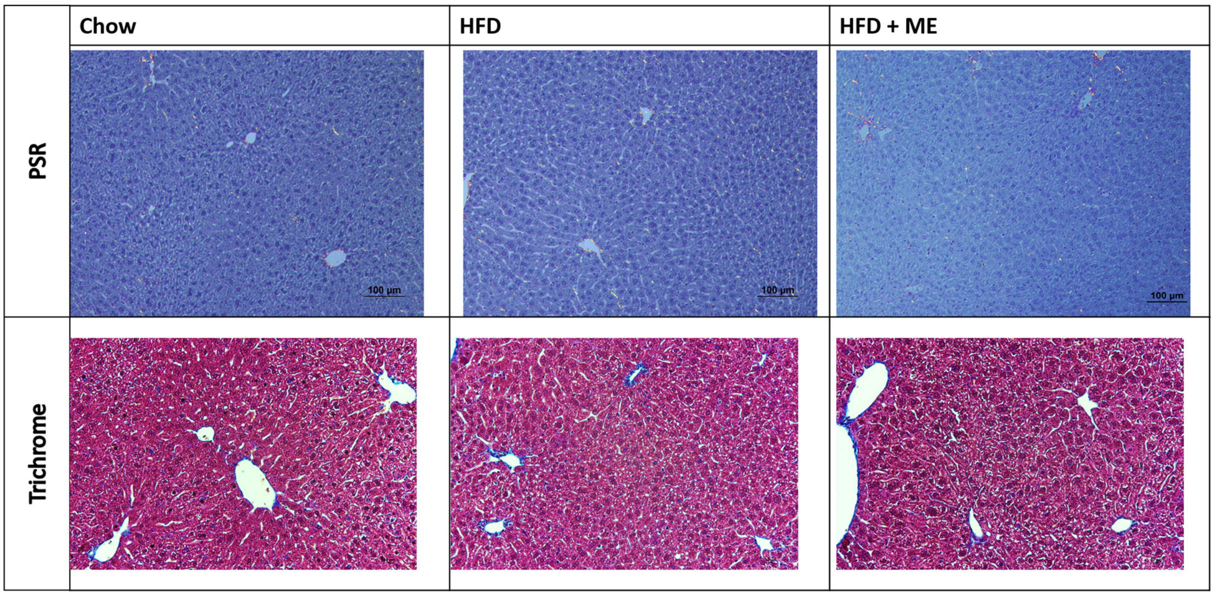

Supplemental Figure S2: PSR and Trichrome.
Mouse livers were stained to assess fibrosis using PSR and trichrome (n=6). These images are representative of a series of images taken from each mouse to allow for statistical comparisons, which were conducted with unpaired Students t-tests using Prism 6 (GraphPad).

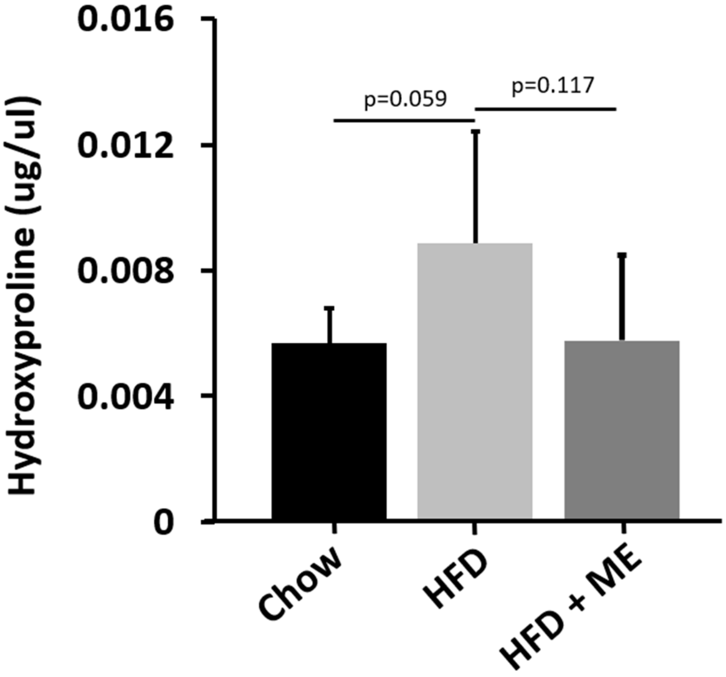

Supplemental Figure S3: Hydroxyproline ELISA.
Hyrdoxyproline ELISA in mouse serum (n=6). All data are shown as mean ±SD. Statistical comparisons conducted with unpaired Students t-tests using Prism 6 (GraphPad).

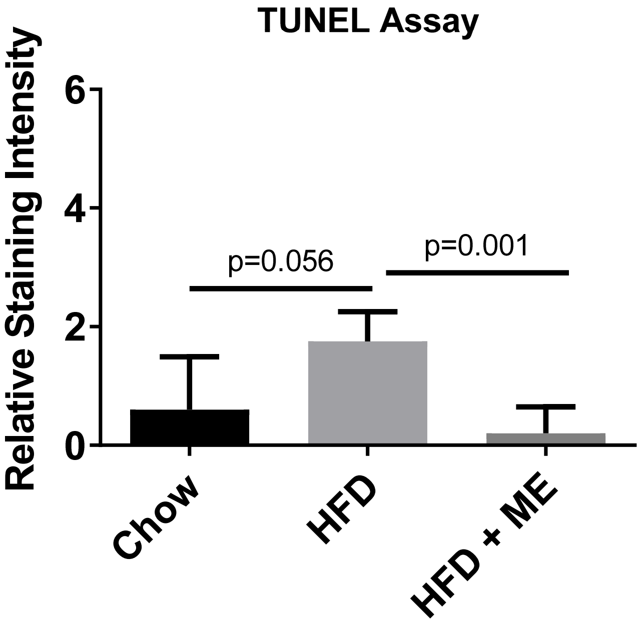

Supplemental Figure S4: TUNEL assay quantification.
Quantification of TUNEL assay in mouse liver (n=5). All data are shown as mean ±SD. Statistical comparisons conducted with unpaired Students t-tests using Prism 6 (GraphPad).

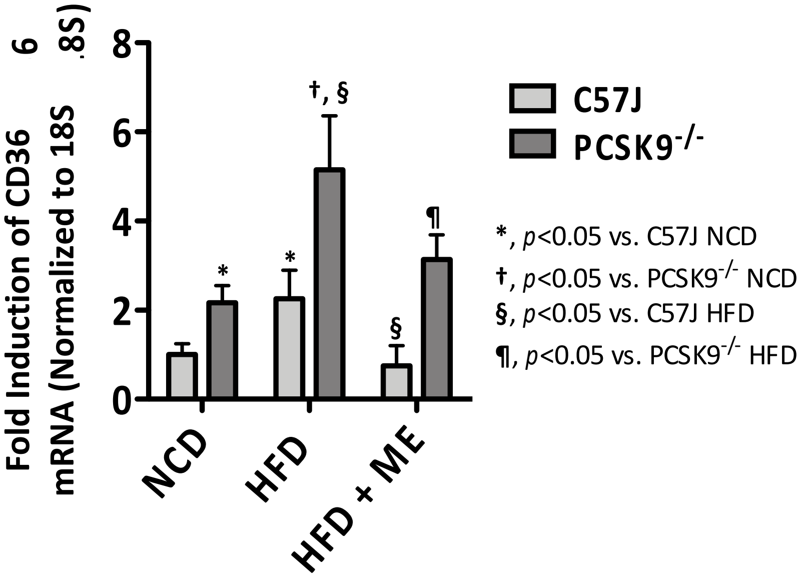

Supplemental Figure S5: Metabolic enhancer attenuates CD36 Expression.
Quantitative real time PCR analysis of mRNA hepatic abundance of CD36 (n=5-6). All data are shown as mean ±SD. *, $p<0.05$ by one-way ANOVA with Tukey multiple comparison testing using Prism 6 (GraphPad).
